# Tobacco Rotated with Rapeseed for Soil-Borne *Phytophthora* Pathogen Biocontrol: Mediated by Rapeseed Root Exudates

**DOI:** 10.3389/fmicb.2016.00894

**Published:** 2016-06-13

**Authors:** Yuting Fang, Limeng Zhang, Yongge Jiao, Jingjing Liao, Lifen Luo, Sigui Ji, Jiangzhou Li, Kuai Dai, Shusheng Zhu, Min Yang

**Affiliations:** ^1^State Key Laboratory for Conservation and Utilization of Bio-Resources in Yunnan, Yunnan Agricultural University Kunming, China; ^2^Key Laboratory for Agro-biodiversity and Pest Control of Ministry of Education, Yunnan Agricultural University Kunming, China; ^3^Yunnan Tobacco Company, Yuxi Branch Yuxi, China

**Keywords:** *Phytophthora parasitica*, root exudates, chemical interaction, soil-borne pathogen, rotation

## Abstract

Black shank, caused by *Phytophthora parasitica* var. *nicotianae*, is a widespread and destructive disease of tobacco. Crop rotation is essential in controlling black shank. Here, we confirmed that rotating black shank-infested fields with rapeseed (*Brassica napus*) suppressed the incidence this disease. Further study demonstrated that rapeseed roots have a strong ability to attract zoospores and subsequently stop the swimming of zoospores into cystospores. Then, rapeseed roots secrete a series of antimicrobial compounds, including 2-butenoic acid, benzothiazole, 2-(methylthio)benzothiazole, 1-(4-ethylphenyl)-ethanone, and 4-methoxyindole, to inhibit the cystospore germination and mycelial growth of *P. parasitica* var. *nicotianae*. Thus, rapeseed rotated with tobacco suppresses tobacco black shank disease through the chemical weapons secreted by rapeseed roots.

## Introduction

Black shank, caused by *Phytophthora parasitica* var. *nicotianae*, is a destructive disease of tobacco (*Nicotiana tabacum* L.), with losses in individual fields reaching 100% (Gallup et al., 2006; Sun et al., 2011). *P. parasitica* var. *nicotianae* is one type of soil-borne pathogen that is difficult to control due to its ability to survive in the soil for many years, even in the absence of tobacco (Erwin and Ribeiro, 1996; Gallup et al., 2006; Sun et al., 2011). Although the use of resistant cultivars, fungicides, and other cultural methods have been recommended to control black shank disease, few of these control measures are sufficiently effective, practical, or economical (Kong et al., 1995; Gallup et al., 2006). Once a soil becomes heavily infested with *P. parasitica* var. *nicotianae*, even the most resistant varieties may be damaged (Kong et al., 1995; Gallup et al., 2006; Sun et al., 2011).

Crop rotation may be the best, most widely practiced, and most cost-effective method for reducing black shank disease and improving tobacco productivity (Gallup et al., 2006; Li et al., 2006; Zhang et al., 2015). Crop rotations can increase soil fertility, soil tilth and aggregate stability; improve soil water management; and reduce erosion and the build-up of soil-borne plant pathogens (Larkin et al., 2010). Previous studies have shown that the rotation of Gramineae, *Brassica*, and *Allium* crops with tobacco successfully suppressed black shank disease of tobacco (Sullivan et al., 2005; Gallup et al., 2006; Zhang et al., 2015). In particular, *Brassica* crops have been the most consistent and effective rotation crops for reducing soil-borne diseases, which infected by *Fusarium* spp., *Sclerotium* spp., *Rhizoctonia* spp., *Streptomyces* spp (Brown and Morra, 1997; Smolinska and Horbowicz, 1999; Matthiessen and Kirkegaard, 2006; Larkin and Griffin, 2007; Larkin, 2008; Larkin et al., 2010) and improving soil characteristics and crop yield (McGuire, 2003). Current production practices in many tobacco production areas of southern China are also based on rotation with rapeseed (*Brassica napus* L.) to control diseases and increase the yield of tobacco (Li et al., 2006). In this area, tobacco is grown from April to October, and rapeseed is subsequently grown from October to March of the next year. Such rotations with rapeseed have been observed to reduce the incidence or severity of some soil-borne tobacco diseases, including black shank (*P. parasitica* var. *nicotianae*), black root rot (*Thielaviopsis basicola*), and brown spot (*Alternaria alternata*), relative to continuous tobacco planting (Li et al., 2006).

Reportedly, crop rotation can help reduce soil-borne pathogens by interrupting the host–pathogen cycle, inhibiting pathogen growth directly, or altering the soil characteristics (Kheyrodin, 2011; Ratnadass et al., 2012; Larkin, 2015). Many studies have investigated the mechanism of soil-borne disease suppression by *Brassica* crops, mainly focusing on biofumigation of green biomass through the production of toxic sulfur metabolites (such as isothiocyanates) and the alteration of soil microbial communities (Sarwar et al., 1998; Mazzola et al., 2001; Cohen et al., 2005; Larkin and Griffin, 2007). However, rapeseed plants grow in the field for approximately 5 months, and the biomass does not totally get incorporated into the soil after harvest in South China. Thus, rapeseed rotated with tobacco for soil-borne disease suppression maybe mainly due to the secretion of antimicrobial substances by rapeseed roots. Plant roots can continuously produce and secrete many compounds into the rhizosphere to mediate the interactions between the roots and pathogens (Bais et al., 2006; Yang et al., 2014). Pathogens can recognize the signals in the root exudates to colonize the host plant (Bais et al., 2004). Plant roots can also secrete a number of substances to protect themselves against pathogen and non-host pathogen infection (Bais et al., 2004, 2006). Thus, it is interesting to investigate whether rapeseed plants growing in the soil can secrete root exudates to help tobacco plants suppress soil-borne diseases.

Here, we aim to: (i) confirm the phenomena of tobacco black shank suppression in a four-year rotation field study with tobacco and rapeseed, (ii) observe the interaction between roots and zoospores, and (iii) determine the mechanisms involved in soil-borne *Phytophthora* disease suppression in tobacco, including the antimicrobial compounds identification in rapeseed root exudates and their antimicrobial activity assessment.

## Materials and Methods

### Field Experiment with Rapeseed and Tobacco Rotation for *P. nicotianae* Suppression

The field rotation study was carried out at the Zhao Wei base of scientific research in Yuxi city (24.497°N, 24.317°E) from 2012 through 2015 to examine the effect that rapeseed and tobacco rotation have on the disease severity of black shank disease in tobacco. The field selected for the study was heavily infested with black shank and contained sandy loam soil. The experiment included two treatments. One treatment involved the rotation of tobacco and rapeseed in which tobacco (cv. KRK26) was grown from May to October and rapeseed (cv. YHY-2) was subsequently grown from October to April of the next year. The other treatment involved continuous tobacco (cv. KRK26) cropping, in which only tobacco was grown from April to October. Each treatment contained three plots (200 m^2^) arranged in the same field using a completely randomized block design. The KRK26 tobacco variety, which is susceptible to *P. parasitica* var. *nicotianae*, was used in this study. During the first week of May each year, healthy greenhouse-grown seedlings with nine leaves were purchased from a market and transplanted in the field. The tobacco plants were planted 0.5 m apart in a row and 1.1 m between rows. Rapeseed variety YHY-2 was used for rotation. The rapeseed plants were transplanted at the seedling age of 30–35 days in October, with a density of 10,000 seedlings/ha. The field management with respect to water, fertilizers, weed control, and insect pest control was uniform for each plot based on the recommendations set forth in the tobacco production guide of Yunnan. The diseased plants with the symptoms of black shank were counted in each plot at the mature stage. Disease incidence = (The number of diseased plants with black shank symptoms in each plot ÷ The total number of plants in each plot) × 100%. Yield data were collected for all plots throughout the four-year period of the experiment. The actual yield per plot was determined as the dry leaf weight of tobacco.

### Effect of Rapeseed Roots on Zoospore Swimming and Cystospore Formation and Germination

Isolate of *P. parasitica* var. *nicotianae* (YXCJ-1, Genbank accession number: KX268718) was collected from tobacco plants with typical symptoms of black shank. YXCJ-1 was grown on carrot agar (CA) medium, and zoospores were produced as described previously (Morris and Ward, 1992). A modified capillary root model, as described by Yang et al. (2014), was used to monitor the interaction of roots and zoospores. Briefly, a capillary tube (1 mm external diameter) was bent into a U-shape, placed on a glass slide and overlaid with a coverslip to form a chamber with one open side. The primary roots of rapeseed (cv. YHY-2) plants were excised with a sterile razor blade, and the root tip was inserted into the open end of the chamber; zoospore suspensions (1 × 10^4^ zoospores/mL) were then added into the chamber. The slides were incubated in a humid petri dish at room temperature. The behavior of zoospores on the root tip and hair zone was recorded every 5 min for a period of 120 min using a video camera attached to a compound microscope (Leica DM2000, Wetzlar, Germany). A capillary tube was inserted into a chamber with the same zoospore suspension as a control. The number of zoospores and cystospores on the different root zones were counted on the photographs from 5 to 25 min. The chemotactic ratio (CR) was determined following the formula described by Muehlstein et al. (1988), where CR = (scores of zoospores and cystospores on the test root)/(score of zoospores and cystospores on the control). Positive CR values indicate positive chemotaxis. The number of germinated and ruptured cystospores as well as the growth direction of the germ tube was recorded on the photographs from 30 to 120 min. The experiment was repeated three times, and six roots were tested per run.

### Root Exudates Collection and Identification

#### Root Exudates Collection

Root exudates of rapeseed (cv. YHY-2) were collected by a previously described trapping system with a few modifications (Yang et al., 2014). Briefly, rapeseed seeds were sterilized with 6% H_2_O_2_ (Sigma–Aldrich Co., Beijing, China) for 8 min and sowed into washed silica sand in glass pots (2 L of sand per pot). Three seeds were sowed into each pot in a greenhouse and irrigated with 0.1-strength Hoagland solution at a rate of 10 mL/day. Additional distilled H_2_O was supplied as needed. When the rapeseed plants reached the six-leaf stage, each pot was washed with 2 L of distilled H_2_O. A column filled with Amberlite XAD-4 resin (Sigma–Aldrich Co., Beijing, China) and fitted with a circulating attachment was then connected to the trapping systems. The solution was circulated at a rate of 1 L/h by airlifting. The root exudates from each column were collected in separate columns, with nine columns in total. The column was detached after 7 days, washed with 10-bed volumes of distilled H_2_O, and then eluted with 200 mL of high-performance liquid chromatography (HPLC) grade methanol (Fisher Scientific, Shanghai) followed by 100 mL of HPLC grade dichloromethane (Fisher Scientific, Shanghai). Eluates from three columns were pooled into one bottle, filtered and concentrated under reduced pressure. The concentrate was dissolved into 1 mL of methanol for further analysis. Control pots without rapeseed plants were treated identically.

#### Gas Chromatography–Mass Spectrometry (GC–MS) Analyses of Root Exudates

The GC–MS fingerprints of the root exudates were obtained on an Agilent 7890-5975 instrument (Agilent, USA). The root exudates were dried under nitrogen gas, followed by methoximation (Sigma–Aldrich Co., Beijing) and trimethylsilylation (Sigma–Aldrich Co., Beijing) derivatization as described by Xu et al. (2012). The root exudates were separated on an HP-5 MS capillary column (19091S-433, 30 m × 0.25 mm × 0.25 μm, Agilent). The injection volume was 1 μL in the splitless mode, and the injector temperature was 260°C. The initial column temperature was 40°C (held 2 min) and programmed to increase at a rate of 5°C/min to 250°C, where it was then held for 10 min. The transfer line temperature was 280°C. Helium (99.999% purity) was used as the carrier gas at a flow rate of 1 mL/min. Mass spectra were obtained in electron impact (EI) ionization mode at 70 eV by monitoring the full-scan range (*m/z* 50–550). The compounds were identified by matching the mass spectra obtained with those of the reference compounds stored in the Wiely7n.1 Library. Components with more than an 80% similarity were regarded as undoubtedly existing in the root exudates. The collection from the control pot was analyzed under similar conditions. The components that appeared in the control treatment were not recorded in the final result.

#### High-Performance Liquid Chromatography (HPLC) Analysis of Root Exudates

The standards of 17 putative compounds identified by GC–MS were purchased from the Guizhou Dida Biological Technology Co. for antimicrobial activity analysis and then eight compounds with antimicrobial activity (Supplementary Table S1) were further selected to determine their concentration in the rapeseed root exudates by HPLC on an Agilent 1260 Infinity instrument (Agilent, USA). The HPLC separations were performed on an Kinetex-C18 column (4.6 × 100 mm, 2.6u) (Phenomenex, Guangzhou) with the following solvent system: solvent A = HPLC grade methanol (Fisher Scientific, Shanghai) and solvent B = 10% methanol and 0.1% phosphoric acid HPLC grade (Sigma–Aldrich Co., Beijing) in HPLC grade water (Fisher Scientific, Shanghai). A multistep gradient was used for all separations with an initial injection volume of 10 μL and a flow rate of 0.5 mL/min. The multistep solvent gradient was as follows: 0–7 min consisted of 22–58% (v/v) solution A, 7–20 min consisted of 58–95% (v/v) solution A, and 20–25 min consisted of isocratic conditions of 95% solution A. The column temperature was maintained at 30°C. Chromatograms were recorded at 210 and 254 nm, and the retention times of the target compounds were established from standards. The compounds in the samples were identified by comparing the results to authentic standards. The concentrations of the target compounds in the samples were quantified using standard curves that showed the linear relationships between the peak areas and the concentrations.

### Inhibition of *P. parasitica* var. *nicotianae* by Rapeseed Root Exudates and Pure Compounds

The inhibitory activity of the root exudates and target compounds on the mycelial growth of *P. parasitica* var. *nicotianae* was determined according to a previously published method (Zhu et al., 2007). Briefly, a fresh plug (5 mm in diameter) was removed from the growing edge of a CA medium culture and transferred onto CA medium supplemented with the root exudates (0, 0.2, 0.4, 0.6, 0.8, and 1.0 mg/mL) or target compounds. All tested compounds were purchased from the Guizhou Dida Biological Technology Co. (Supplementary Table S1) and were each dissolved in methanol (Fisher Scientific, Shanghai) to prepare stock solutions, which were diluted in distilled water to the test concentrations. The concentrations of all target compounds in the CA medium are listed in Supplementary Table S1. The antimicrobial activity of target compounds were preliminary assessed on CA medium amended with compound at concentration of 0, 400, 600, 800, and 1000 mg/L. If the compound showed significant antimicrobial activities, the concentrations, which inhibit the mycelial growth of *P. parasitica* var. *nicotianae* from 10 to 90%, were further determined. In all cases, the final amount of solvent never exceeded 1% (vol/vol) in the treated and control samples. Mycelial growth was assessed by measuring the colony diameter after dark incubation at 25°C for 4 days.

The inhibitory activity of the root exudates and target compounds against the zoospore motility and cystospore germination was measured according to a published procedure with slight modifications (Yang et al., 2014). Briefly, an aliquot of 40 μL of a target compound solution was added to depression glass slides. Then, 40 μL of a zoospore suspension (1 × 10^5^ zoospores/mL) or cystospore suspension (1 × 10^5^ cystospores/mL) was added immediately to the glass slides containing each respective solution. The slides were placed in Petri dishes containing moist filter paper and incubated in the dark at 24°C. The percentage of zoospores encysted into cystospores was recorded under the microscope after zoospore incubation for 20 min. The number of germinated cystospores was counted under a microscope after cystospore incubation for 4 h. The experiment was conducted three times, each time in triplicate.

The inhibition of mycelial growth, zoospore motility and cystospore germination by the root exudates or target compounds was calculated (Zhu et al., 2007). The inhibition rate (%) = 100% × (*D*_control_ - *D*_treated_)/*D*_control_, in which *D*_control_ is the expansion diameter of mycelia on media without compound (0 mg/mL), *D*_treated_ is the expansion diameter of mycelia on the media amended with different concentrations of compounds. The median effective concentration value (EC_50_) for each isolate was calculated by regressing the percentage of growth inhibition against the logarithm value of the fungicide concentration using the software Microsoft Excel 2003.

### Data Analysis

All data from the different treatments were evaluated by one-way analysis of variance (ANOVA) followed by Fisher’s least significant difference (LSD) test (*p* < 0.05) with PASW Statistics 18 (SPSS Inc., Chicago, IL, USA).

## Results

### Rapeseed and Tobacco Rotation Suppressed Black Shank in Tobacco

**FIGURE 1:**
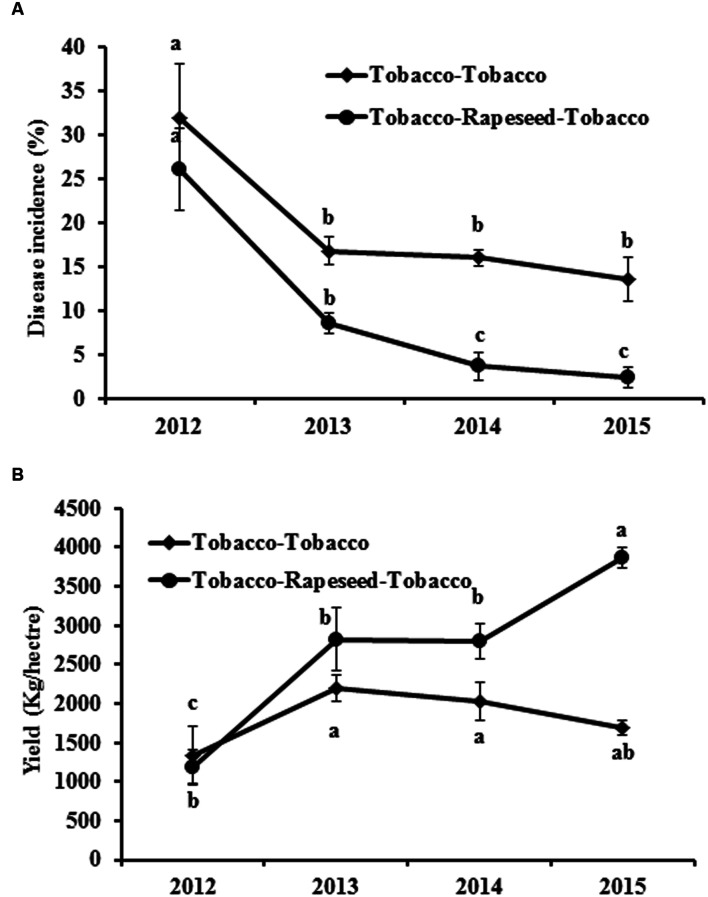
**Effect of rapeseed and tobacco rotation on black shank disease in tobacco and tobacco yield from 2012 through 2015. (A, B)** Show the black shank disease incidence and the leaf yield in continuous tobacco cropping fields or in tobacco and rapeseed rotation fields, respectively. Bars represent the means ± SE. Bars with different letters are significantly different (*p* < 0.05).

The disease incidence of black shank in the field ranged from 25 to 35% in 2012 (**Figure 1A**). After rotation with rapeseed, the disease incidences significantly decreased compared with the continuous tobacco cropping (**Figure 1A**). Although the disease incidences all decreased in both treatments in 2013, the decreased rate in rapeseed rotation treatment reached 67.47%, which higher than 47.41% in continuous tobacco cropping treatment. Notably, the disease incidence gradually decreased with an increased rotation time from 2012 to 2015 (**Figure 1A**). The yield of tobacco also increased 128.8% after rotation with rapeseed compared with the continuous tobacco cropping (**Figure 1B**).

### Rapeseed Roots Interfere with the Behavior and Development of Zoospores

**FIGURE 2:**
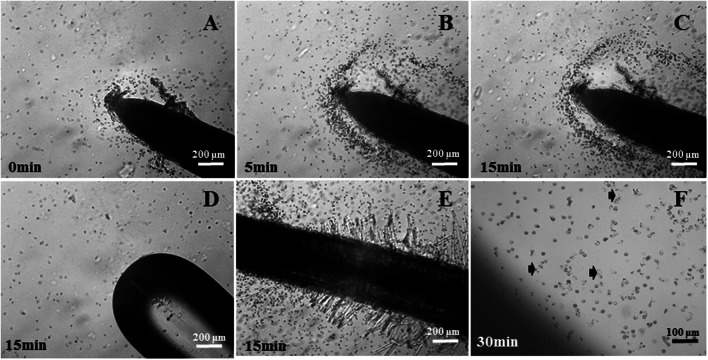
**Interaction analysis of rapeseed roots with zoospores of *Phytophthora parasitica* var. *nicotianae*. (A–C)** Spores were attracted by rapeseed root and clustered in the rhizosphere from 0 to 15 min. **(D)** The chemotactic ability of zoospores towards the capillary tube after incubated for 15 mins. **(E)** The chemotactic ability of zoospores towards the root hair zone after incubated for 15 min. **(F)** The rupture of spores near the root. Arrow in **(F)** shows the ruptured spores.

The zoospores exhibited strong chemotaxis towards the rapeseed roots and then attached to the surfaces of the root tips (**Figures 2A–C**) and the root hair zone (**Figure 2E**) but did not exhibit chemotaxis towards the capillary tube (**Figure 2D**). The CR also indicated that the zoospores exhibited positive chemotaxis toward the rapeseed roots (Supplementary Table S2), which occurred within five minutes after the zoospores were exposed to the rapeseed root (Supplementary Table S2). After being attracted to the rapeseed root surface, the zoospores quickly stopped and encysted into the cystospores on the root surface or near the root (**Figures 2B, C**). Some cystospores on the rapeseed root tips even ruptured after 30 min (**Table 1**; **Figure 2F**). After 120 min incubation, 33.84% of cystospores germinated and 46.11% of the germ tubes grew toward the rapeseed roots, which was significantly higher than in the control treatment (**Table 1**).

**Table 1:** Influence of *Phytophthora parasitica* var. *nicotianae* cystospores with rapeseed roots.

Treatments	Cystospore rupture rate (%)^a^	Cystospore germination rate (%)^b^	Percentage of germ tube growth towards the root (%)^b^
Roots	8.14 ± 2.59**	33.84 ± 10.46**	46.11 ± 9.26**
Capillary tube	0.21 ± 0.21	1.56 ± 0.79	18.59 ± 9.59

*^a^The cystospore rupture rate was calculated at 30 min after zoospores exposure to root*

*^b^The cystospore germination rate and percentage of germ tube growth towards the root were recorded at 120 min after zoospores exposure to root.*

Double asterisks (**) indicate statistically significant differences (Student’s *t*-test; *p* < 0.01; *n* = 15).

### Rapeseed Root Exudates Inhibit the Growth of *P. parasitica* var. *nicotianae*

**FIGURE 3:**
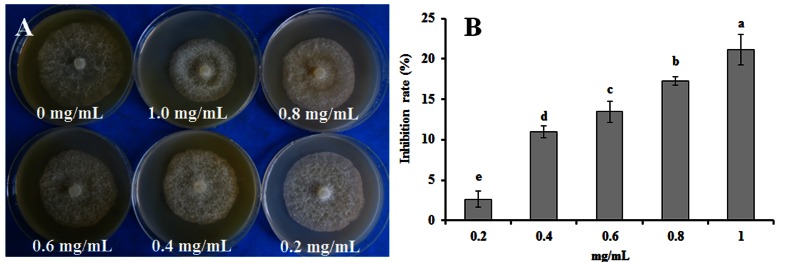
**(A)** Effect of rapeseed root exudates on the mycelial growth of *P. parasitica* var. *nicotianae*. **(B)** Bars are the means ± SE. Bars with different letters are significantly different (*p* < 0.05).

The rapeseed root exudates had dose-dependent antimicrobial activity against the mycelial growth of *P. parasitica* var. *nicotianae* (**Figure 3**). The inhibition rate reached 21.09% when mycelia were exposed to the root exudates at concentration of 1.0 mg/mL (**Figure 3**).

### Compound Identification in the Root Exudates

Gas chromatography–mass spectrometry analysis identified a total of 26 putative compounds in the rapeseed root exudates (Supplementary Figure S1; **Table 2**), which were identified as 6 acids, 4 esters, 2 ketones, 2 aldehydes, 3 nitrogen-containing compounds, 8 alkanes, and 1 indole based on their chemical nature.

**Table 2:** Compounds identified by GC–MS analysis in rapeseed root exudates.

Group	Peak	Closest compound	Formula	Molecular weight	Characteristic fragments	Spectra similarity (%)^a^
Acids	3	2-Butenoic acid	C_4_H_6_O_2_	86	86, 69, 57, 53, 49, 45	91
	4	Valeric acid	C_5_H_10_O_2_	102	73, 60, 55, 45, 43, 42, 41, 39	83
	6	2-Pentenoic acid	C_5_H_8_O_2_	100	100, 55, 57, 45, 43, 41, 39	90
	15	Nonanoic acid	C_9_H_18_O_2_	158	129, 115, 98, 73, 60, 55	80
	22	*n*-Hexadecanoic acid	C_16_H_32_O_2_	256	256, 213, 129, 73, 43	98
	21	9-Hexadecenoic acid	C_16_H_30_O_2_	254	254, 236, 97, 83, 69, 55, 41	96
Esters	2	Methyl thiocyanate	C_2_H_3_NS	73	73, 58, 49, 47, 45	90
	7	Cyclohexyl isocyanate	C_7_H_11_NO	125	125, 97, 82, 67, 41	93
	13	Cyclohexyl isothiocyanate	C_7_H_11_NS	141	141, 83, 55	87
	24	Linoleic acid ethyl ester	C_20_H_36_ O_2_	308	308, 263, 123, 109, 95, 81, 67, 55, 41	99
Ketones	5	2-Heptanone	C_7_H_14_O	114	114, 99, 71, 58, 43, 39	80
	14	1-(4-Ethylphenyl)-ethanone	C_10_H_12_O	148	148, 133, 105,	97
Aldehydes	1	3-Methyl butyraldehyde	C_5_H_10_O	86	86, 71, 58, 55, 44, 41, 38	86
	10	Nonanal	C_9_H_18_O	142	124, 119, 98, 82, 57, 41	80
Nitrogen-containing compounds	12	Benzothiazole	C_7_H_5_NS	135	135, 108, 91	83
	19	2-(Methylthio)benzothiazole	C_8_H_7_NS_2_	181	181, 148, 136, 108, 69	95
	26	9-Octadecenamide	C_18_H_35_NO	281	281, 126, 98, 72, 59, 41	99
Alkanes	8	Decane	C_10_H_22_	142	142, 113, 85, 71, 57, 43	94
	9	Undecane	C_11_H_24_	156	156, 85, 71, 57, 43, 39	90
	11	Dodecane	C_12_H_26_	170	170, 85, 71, 57, 43, 39	94
	16	Tetradecane	C_14_H_30_	198	198, 155, 85, 71, 57, 43	96
	18	Hexadecane	C_16_H_34_	226	226, 85, 71, 57, 43	87
	20	Octadecane	C_18_H_38_	254	254, 99, 85, 71, 57, 43	98
	23	Eicosane	C_20_H_42_	282	282, 99, 85, 71, 57	98
	25	1-Docosene	C_22_H_44_	308	125, 111, 97, 83, 69, 55, 43	99
Indoles	17	4-Methoxyindole	C_9_H_9_NO	147	147, 132, 104	94

*^a^Spectra similarity compared with mass spectrum of the reference compounds stored in Wiley71.1 (%).*

### Inhibitory Activity Against *P. parasitica* var. *nicotianae* of the Compounds in the Root Exudates

**FIGURE 4:**
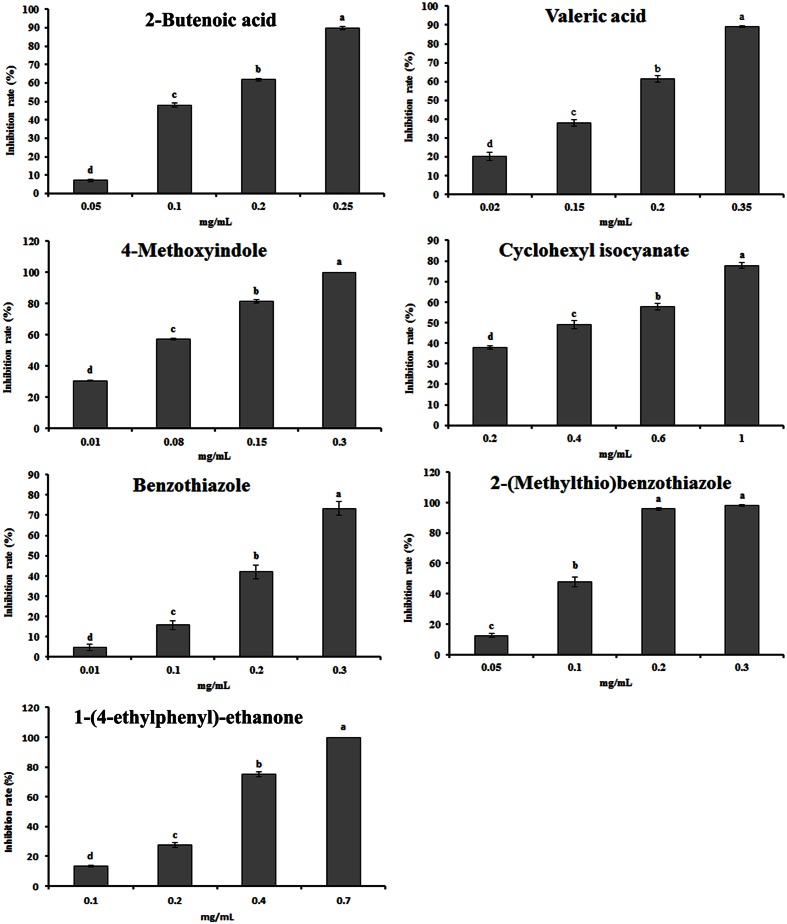
**Effect of the identified compounds in root exudates on the mycelial growth of *P. parasitica* var. *nicotianae*.** Bars are the means ± SE. Bars with different letters are significantly different (*p* < 0.05).

The antimicrobial activity of 14 compounds in the rapeseed root exudates was tested using pure compounds. Among these compounds, 2-butenoic acid, valeric acid, 4-methoxyindole, cyclohexyl isocyanate, benzothiazole, 2-(methylthio)benzothiazole and 1-(4-ethylphenyl)-ethanone showed significant dose-dependent antimicrobial activity against the growth of mycelia (**Figure 4**). The EC_50_ values ranged from 27.46 to 366.37 mg/L (Supplementary Table S1). Other seven compounds, such as several alkanes and palmitic acid, did not have significant antimicrobial activity (Supplementary Table S1).

### Compound Concentrations in the Root Exudates and Their Antimicrobial Activities

**FIGURE 5:**
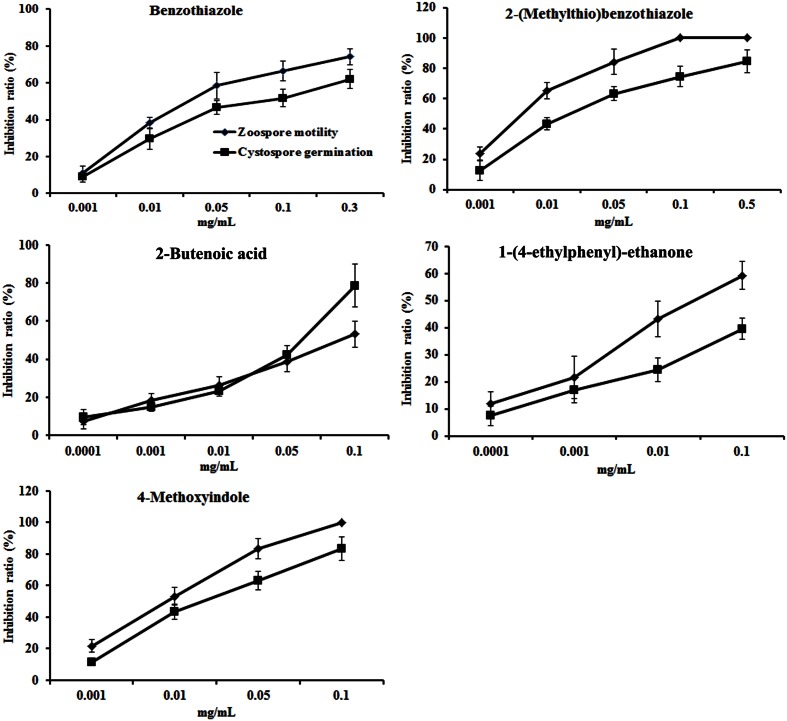
**The inhibitory activity of compounds against the motility of zoospores and the germination of cystospores.** Error bars indicate the SE of three replicates.

The concentrations of the above seven antimicrobial compounds in the rapeseed root exudates were further analyzed by HPLC. Only 2-butenoic acid, benzothiazole, 2-(methylthio)benzothiazole, 1-(4-ethylphenyl)-ethanone, and 4-methoxyindole were quantified by HPLC (**Table 3**; Supplementary Figure S2). The antimicrobial activity of these compounds against zoospore motility and cystospore germination of *P. parasitica* var. *nicotianae* was further tested based on their concentration in the root exudates. These five compounds exerted dose-respondent inhibitory effects on zoospore motility and cystospore germination (**Figure 5**). The EC_50_ values of these five compounds for zoospore motility and cystospore germination were 0.64–133.83 and 3.28–523.96 mg/L, respectively. All of these compounds showed high activity against zoospore motility compared with cystospore germination (**Figure 5**).

**Table 3:** Concentration of the target compounds in rapeseed root exudates from HPLC.

Compound	Calibration curve	Concentration (μg/mL) ± standard error (*SE*)
2-Butenoic acid	*Y* = 0.0206x - 6.8416, *R*^2^ = 0.9971	0.76 ± 0.32
Benzothiazole	*Y* = 0.0122x - 2.2555, *R*^2^ = 0.9997	10.02 ± 2.45
2-(Methylthio)benzothiazole	*Y* = 0.0240x - 1.3610, *R*^2^ = 0.9999	7.14 ± 2.67
1-(4-ethylphenyl)-ethanone	*Y* = 0.0181 + 1.0301, *R*^2^ = 0.9999	2.18 ± 0.97
4-Methoxyindole	*Y* = 0.0125x - 3.9263, *R*^2^ = 0.9988	56.54 ± 10.28

## Discussion

Tobacco monoculture leads to outbreaks of black shank in tobacco and decreased yields (Kong et al., 1995; Gallup et al., 2006). Our 4-year field experiment confirmed that rapeseed and tobacco rotation significantly suppressed black shank disease in tobacco. Many other studies also support the high potential for using rapeseed in rotation, cover, or green manure crops for the suppression of soil-borne diseases (Larkin and Griffin, 2007; Larkin et al., 2010). Reportedly, crop rotation can help reduce soil-borne pathogens, such as fungi, bacteria, Oomycetes, and nematodes, by (i) interrupting or breaking the host-pathogen cycle; (ii) altering the soil characteristics to make the soil environment less conducive for pathogen development or survival, often stimulating microbial activity and diversity or beneficial plant microbes; or (iii) directly inhibiting pathogens either through the production of inhibitory or toxic compounds in the roots or plant residues or the stimulation of specific microbial antagonists that directly suppress the pathogen inoculum (Larkin et al., 2010; Larkin, 2015).

In the present study, rapeseed root exudates play important role in the suppression of *P. parasitica* var. *nicotianae*. This pathogen is a typical soil-borne pathogen that infects plants through the production of zoospores, which involves a pre-penetration process of zoospore taxis, encystment, cystospore germination, and orientation of the germ tube (Erwin and Ribeiro, 1996). We found that rapeseed roots could attract zoospores to their surface where they were encysted into cystospores. After the cystospores germinated, the growth of the germ tube also proceeded toward the roots. Some studies have indicated that the attraction of plant roots to zoospores was not host specific (Deacon, 1988). The chemotaxis and electrotaxis of zoospores toward plant roots are involved in the attraction of zoospores to host and non-host roots (Cameron and Carlile, 1978; Carlile, 1983; van West et al., 2002). After being attracted by the rapeseed roots, some of the spores could not germinate, and some spores even ruptured, which indicates that rapeseed roots can attract zoospores and hyphal growth and then secrete antimicrobial substances against the infection by the zoospores and hyphal growth.

Evidently, rapeseed root exudates showed dose-dependent antimicrobial activity against the mycelial growth of *P. parasitica* var. *nicotianae*. A library of antimicrobial compounds, including 2-butenoic acid, valeric acid, 4-methoxyindole, cyclohexyl isocyanate, benzothiazole, 2-(methylthio)benzothiazole and 1-(4-ethylphenyl)-ethanone, were further identified by GC–MS in the rapeseed root exudates. These compounds showed significant dose-dependent antimicrobial activity against zoospore motility, cystospore germination, and mycelia growth. Notably, 2-butenoic acid, benzothiazole, 2-(methylthio)benzothiazole, 1-(4-ethylphenyl)-ethanone, and 4-methoxyindole showed antimicrobial activity against zoospore motility and cystospore germination at the concentrations detected in the rapeseed root exudates. Among these compounds, benzothiazole and 2-(methylthio)-benzothiazole have previously been identified as antimicrobial compounds against some fungal and oomycete phytopathogens in the root exudates and volatiles of plants as well as in soil bacteria (Bjostard and Hibbard, 1992; Sun et al., 2003; Lane et al., 2004; He et al., 2005; Gaquerel et al., 2009; Hu et al., 2009; Yadav et al., 2011; Zhang et al., 2011; Xu et al., 2012; Yang et al., 2014). 2-Butenoic acid and valeric acid are important organic acids in plant root exudates and possess antimicrobial activity (Corsetti et al., 1998; Baudoin et al., 2003; Sandnes et al., 2005). 1-(4-ethylphenyl)-ethanone exhibited antimycobacterial activity (Rajabi et al., 2005), and 4-methoxyindole and other indole derivatives have been reported in root exudates with antimicrobial activity (Seal et al., 2004; Paudel et al., 2012; Wu and Chen, 2015). The *Brassicaceae* family produces sulfur compounds that break down to produce isothiocyanates that are toxic to many soil organisms (Sarwar et al., 1998). In this study, cyclohexyl isocyanate was identified in the root exudates and showed antimicrobial activity. Although the antimicrobial activity of these compounds in rapeseed root exudates was identified, the secretion mechanism of these compounds from root tissue and their mode of action against *P. parasitica* var. *nicotianae* are still unknown.

The above data demonstrated that rapeseed can secrete many antimicrobial compounds through its root exudates to kill soil-borne pathogens. Plants are a primary driver of changes in soil microbial communities, and recent studies have documented that crop rotations can dramatically affect these communities (Lupawayi et al., 1998; O’Donnell et al., 2001; Larkin, 2003, 2008; Sturz and Christie, 2003; Larkin and Griffin, 2007; Van Elsas and Costa, 2007; Larkin et al., 2010; Chaparro et al., 2012) and stimulate specific microbial antagonists that directly suppress pathogen inocula (Mazzola et al., 2001; Garbeva et al., 2004; Welbaum et al., 2004). *Brassica* crops rotated with potato had higher microbial activity and diversity, which may help to suppress soil-borne diseases potatoes (Garbeva et al., 2004; Welbaum et al., 2004; Ghorbani et al., 2008; Larkin, 2008). Thus, whether rotating rapeseed with tobacco increases the soil microbial activity and diversity, the populations of plant-beneficial organisms, and the antagonism toward pathogens to result in disease suppression is interesting and should be evaluated further.

## Conclusion

Rapeseed rotated with tobacco can effectively suppress black shank disease of tobacco caused by *P. parasitica* var. *nicotianae* in the field. Rapeseed roots can attract zoospores into the rhizosphere and then secrete a series of antimicrobial substances to kill them, which eventually caused *P. parasitica* var. *nicotianae* to lose its ability to spread or survive in the soil.

## Author Contributions

SZ, LZ and MY designed the research; YF, YJ, JL, LL, and JL performed the research; KD, SJ, and LZ analyzed the data; SZ and MY wrote the paper; LZ, YJ and SJ reviewed the paper.

## Conflict of Interest Statement

The authors declare that the research was conducted in the absence of any commercial or financial relationships that could be construed as a potential conflict of interest.

## References

[B1] Bais H. P. Park S. W. Weir T. L. Callaway R. M. Vivanco J. M. (2004). How plants communicate using the underground information superhighway. *Trends Plant Sci.* 9, 26–32. 10.1016/j.tplants.2003.11.008 14729216

[B2] Bais H. P. Weir T. L. Perry L. G. Gilroy S. Vivanco J. M. (2006). The role of root exudates in rhizosphere interactions with plants and other. *Annu. Rev. Plant Biol.* 57 233–266. 10.1146/annurev.arplant.57.032905.105159 16669762

[B3] Baudoin E. Benizri E. Guckert A. (2003). Impact of artificial root exudates on the bacterial community structure in bulk soil and maize rhizosphere. *Soil Biol. Biochem.* 35 1183–1192. 10.1016/S0038-0717(03)00179-2

[B4] Bjostard L. B. Hibbard B. E. (1992). 6-Methoxy-2-benzoxazotinone: a semiochemical for host location by Western corn root worm 1arvae. *J. Chem. Ecol.* 18 931–944. 10.1007/BF00980054 24254139

[B5] Brown P. D. Morra M. J. (1997). Control of soilborne plant pests using glucosinolate containing plants. *Adv. Agron.* 61 167–231. 10.1016/S0065-2113(08)60664-1

[B6] Cameron J. N. Carlile M. J. (1978). Fatty acids, aldehydes and alcohols as attractants for zoospores of *Phytophthora palmivora*. *Nature* 271 448–449. 10.1038/271448a0

[B7] Carlile M. J. (1983). “Motility, taxis and tropism in Phytophthora,” in *Phytophthora: Its Biology, Taxonomy, Ecology and Pathology* eds Erwin D. C. Bartnicki-Garcia S. Tsao P. H. (St. Paul, MA: APS Press) 95–107.

[B8] Chaparro J. M. Sheflin A. M. Manter D. K. Vivanco J. M. (2012). Manipulating the soil microbiome to increase soil health and plant fertility. *Biol. Fertil. Soils* 48 489–499. 10.1007/s00374-012-0691-4

[B9] Cohen M. F. Mazzola M. Yamasaki H. (2005). *Brassica napus* seed meal soil amendment modifies microbial community structure, nitric oxide production and incidence of Rhizoctonia root rot. *Soil Biol. Biochem.* 37 1215–1227. 10.1016/j.soilbio.2004.11.027

[B10] Corsetti A. Gobbetti M. Rossi J. Damiani P. (1998). Antimould activity of sourdough lactic acid bacteria: identification of a mixture of organic acids produced by *Lactobacillus sanfrancisco* CB1. *Appl. Microbiol. Biotechnol.* 50 253–256. 10.1007/s002530051285 9763693

[B11] Deacon J. W. (1988). Behavioural responses of fungal zoospores. *Microbiol. Sci.* 5 249–252. 3079187

[B12] Erwin D. C. Ribeiro O. K. (1996). “Phytophthora capsici,” in *Phytophthora Diseases Worldwide* eds Erwin D. C. Ribeiro O. K. (St. Paul, MN: APS Press), 262–268.

[B13] Gallup C. A. Sullivan M. J. Shew H. D. (2006). Black shank of tobacco. *Plant Health Instruct.* 10.1094/PHI-I-2006-0717-01

[B14] Gaquerel E. Weinhold A. Baldwin I. T. (2009). Molecular interactions between the specialist herbivore *Manduca sexta* (Lepidoptera, Sphigidae) and its natural host *Nicotiana attenuata*. VIII. An unbiased GC6GC-ToFMS analysis of the plant’s elicited volatile emissions. *Plant Physiol.* 149 1408–1423. 10.1104/pp.106.088781 19136568 PMC2649405

[B15] Garbeva P. van Veen J. A. van Elsas J. D. (2004). Microbial diversity in soil: selection of microbial populations by plant and soil type and implications for disease suppressiveness. *Annu. Rev. Phytopathol.* 42 243–270. 10.1146/annurev.phyto.42.012604.135455 15283667

[B16] Ghorbani R. Wilockson S. Koocheki A. Leifert C. (2008). Soil management for sustainable crop disease control: a review. *Environ. Chem. Lett.* 6 149–162. 10.1007/s10311-008-0147-0

[B17] He H. B. Chen X. X. Lin R. X. Lin W. X. He H. Q. Jia X. L. (2005). Chemical components of root exudates from allelopathic rice accession PI312777 seedlings, Chinese. *J. Appl. Ecol.* 16 2383–2388. 10.13287/j.1001-9332.2005.0356 16515193

[B18] Hu Z. H. Shen Y. B. Shen F. Y. Luo Y. Q. Su X. H. (2009). Evidence for the signaling role of methyl jasmonate, methyl salicylate and benzothiazole between poplar (Populus simonii 6P. pyramidalis ‘Opera 8277’) cuttings. *Trees* 23 1003–1011. 10.1007/s00468-009-0342-z

[B19] Kheyrodin H. (2011). Crop rotations for managing soil-borne plant diseases. *Afr. J. Food Sci. Technol.* 2 1–9.

[B20] Kong F. Zhu X. Shi J. Guo Z. (1995). Developing tendency causes and control measures of tobacco infectious diseases in China. *China Tobacco* 16 31–34. 10.13496/j.issn.1007-5119.1995.01.008

[B21] Lane N. Weidenhamer J. D. Romeo J. T. (2004). *Zapoteca formosa*: sulfur chemistry and phytotoxicity. *J. Chem. Ecol.* 30 425–437. 10.1023/B:JOEC.0000017986.49513.f7 15112733

[B22] Larkin R. P. (2003). Characterization of soil microbial communities under different potato cropping systems by microbial population dynamics, substrate utilization, and fatty acid profiles. *Soil Biol. Biochem.* 35 1451–1466. 10.1094/PHYTO-04-10-0100

[B23] Larkin R. P. (2008). Relative effects of biological amendments and crop rotations on soil microbial communities and soilborne diseases of potato. *Soil Biol. Biochem.* 40 1341–1351. 10.1016/j.soilbio.2007.03.005

[B24] Larkin R. P. (2015). Soil health paradigms and implications for disease management. *Annu. Rev. Phytopathol.* 53 199–221. 10.1146/annurev-phyto-080614-120357 26002292

[B25] Larkin R. P. Griffin T. S. (2007). Control of soilborne diseases of potato using *Brassica green* manures. *Crop Protect.* 26 1067–1077. 10.1016/j.cropro.2006.10.004

[B26] Larkin R. P. Griffin T. S. Honeycutt C. W. (2010). Rotation and cover crop effects on soilborne potato diseases, tuber yield, and soil microbial communities. *Plant Dis.* 94 1491–1502. 10.1094/PDIS-03-10-0172 30743393

[B27] Li T. Wang B. Wang S. (2006). The current situations, problems and measures of tobacco in Yunnan. *Chinese Tobacco Sci.* 27 48–51. 10.13496/j.issn.1007-5119.2006.02.015

[B28] Lupawayi N. Z. Rice W. A. Clayton G. W. (1998). Soil microbial diversity and community structure under wheat as influenced by tillage and crop rotation. *Soil Biol. Biochem.* 30 1733–1741. 10.1016/S0038-0717(98)00025-X

[B29] Matthiessen J. N. Kirkegaard J. A. (2006). Biofumigation and enhanced biodegradation: opportunity and challenge in soilborne pest and disease management. *Crit. Rev. Plant Sci.* 25 235–265. 10.1080/07352680600611543

[B30] Mazzola M. Granatstein D. M. Elfving D. C. Mullinix K. (2001). Suppression of specific apple root pathogens by *Brassica napus* seed meal amendment regardless of glucosinolate content. *Phytopathology* 91 673–679. 10.1094/PHYTO.2001.91.7.673 18942997

[B31] McGuire A. N. (2003). Mustard green manures replace fumigant and improve infiltration in potato cropping system. *Crop Manag.* 2 10.1094/CM-2003-0822-01-RS

[B32] Morris P. F. Ward E. W. B. (1992). Chemoattraction of zoospores of the soybean pathogen, *Phytophthora sojae*, by isoflavones. *Physiol. Mol. Plant Pathol.* 40 17–22. 10.1016/0885-5765(92)90067-6

[B33] Muehlstein L. K. Amon J. P. Leﬄer D. L. (1988). Chemotaxis in the marine fungus *Rhizophydium littoreum*. *Appl. Environ. Microbiol.* 54 1668–1672. 16347677 10.1128/aem.54.7.1668-1672.1988 PMC202725

[B34] O’Donnell A. G. Seasman M. MacRae A. Waite I. Davies J. T. (2001). Plants and fertilizers as drivers of change in microbial community structure and function in soils. *Plant Soil* 232 135–145. 10.1023/A:1010394221729

[B35] Paudel A. Hamamoto H. Kobayashi Y. Yokoshima S. Fukuyama T. Sekimizu K. (2012). Identification of novel deoxyribofuranosyl indole antimicrobial agents. *J. Antibiot.* 65 53–57. 10.1038/ja.2011.110 22167161

[B36] Rajabi L. Courreges C. Montoya J. Aguilera R. J. Primm T. P. (2005). Acetophenones with selective antimycobacterial activity. *Lett. Appl. Microbiol.* 40 212–217. 10.1111/j.1472-765X.2005.01657.x 15715647

[B37] Ratnadass A. Fernandes P. Avelino J. Habib R. (2012). Plant species diversity for sustainable management of crop pests and diseases in agroecosystems: a review. *Agron. Sustain. Dev.* 32 273–303. 10.1007/s13593-011-0022-4

[B38] Sandnes A. Eldhuset T. D. Wollebaek G. (2005). Organic acids in root exudates and soil solution of Norway spruce and silver birch. *Soil Biol. Biochem.* 37 259–269. 10.1016/j.soilbio.2004.07.036

[B39] Sarwar M. Kirkegaard J. A. Wong P. T. W. Desmarchelier J. M. (1998). Biofumigation potential of brassicas. III. In vitro toxicity of isothiocyanates to soil-borne fungal pathogens. *Plant Soil* 210 103–112. 10.1023/A:1004381129991

[B40] Seal A. N. Pratley J. E. Haig T. An M. (2004). Identification and quantitation of compounds in a series of allelopathic and non-allelopathic rice root exudates. *J. Chem. Ecol.* 30 1647–1662. 10.1023/B:JOEC.0000042074.96036.14 15537165

[B41] Smolinska U. Horbowicz M. (1999). Fungicidal activity of volatiles from selected cruciferous plants against resting propagules of soil-borne fungal pathogens. *J. Phytopathol.* 147 119–124. 10.1046/j.1439-0434.1999.147002119.x

[B42] Sturz A. V. Christie B. R. (2003). Beneficial microbial allelopathies in the root zone: the management of soil quality and plant disease with rhizobacteria. *Soil Tillage Res.* 72 107–123. 10.1016/S0167-1987(03)00082-5

[B43] Sullivan M. J. Melton T. A. Shew H. D. (2005). Managing the race structure of *Phytophthora parasitica* var. nicotianae with cultivar rotation. *Plant Dis.* 89 1285–1294. 10.1094/PD-89-1285 30791306

[B44] Sun H. Y. Wang Y. Z. Yang L. (2003). Analysis of the major components of root exudates released from several economic forest tree using GC-MS. *J. For. Res.* 14 127–129. 10.1007/BF02856778

[B45] Sun J. Li X. Wu Z. Sun H. (2011). Progress on tobacco black shank disease. *Hubei Agric. Sci.* 50 3253–3255. 10.14088/j.cnki.issn0439-8114.2011.16.030

[B46] Van Elsas J. D. Costa R. (2007). “Molecular assessment of soil microbial communities with potential for plant disease suppression,” in *Biotechnology and Plant Disease Management* eds Punja Z. K. Boer S. H. Sanfacon H. (King’s Lynn: CABI), 498–517.

[B47] van West P. Morris B. M. Reid B. Appiah A. Osborne M. C. Campbell T. A. (2002). Oomycete plant pathogens use electric fields to target roots. *Mol. Plant Microbe Interact* 15 790–798. 10.1094/MPMI.2002.15.8.790 12182336

[B48] Welbaum G. E. Sturz A. V. Dong Z. Nowak J. (2004). Managing soil microorganisms to improve productivity of agroecosystems. *Crit. Rev. Plant Sci.* 23 175–193. 10.1080/07352680490433295

[B49] Wu Y. Chen G. (2015). Progress of the antimicrobial activities of indole-2,3-dione derivatives. *Chem. Reagents* 37 702–706. 10.13822/j.cnki.hxsj.2015.08.007

[B50] Xu N. Wang C. Wei M. Shi W. Wang X. F. (2012). Allelopathy of Welsh onion root exudates on cucumber seed germination and *Fusarium oxysporum* f. sp. cucumerinum and the GC-MS analysis. *Acta Hortic. Sin.* 39 1511–1520.

[B51] Yadav P. S. Devprakash Senthilkumar G. P. (2011). Benzothiazole: different methods of synthesis and diverse biological activities. *Int. J. Pharm. Sci. Drug Res.* 3 1–7. 10.1002/chin.201140238

[B52] Yang M. Zhang Y. Qi L. Mei X. Liao J. Ding X. (2014). Plant-plant-microbe mechanisms involved in soil-borne disease suppression on a maize and pepper intercropping system. *PLoS ONE* 9:e115052 10.1371/journal.pone.0115052 PMC4281244 25551554

[B53] Zhang L. Fang Y. Ji S. Jiao Y. Liao J. Li J. (2015). Inhibitory activity of maize root exudates against *Phytophthora nicotianae* and antifungal compounds analysis. *Chinese J. Biol. Control* 31 115–122. 10.16409/j.cnki.2095-039x.2015.01.016

[B54] Zhang S. H. Sun P. S. Ge F. J. Wu Z. B. (2011). Different sensitivities of *Selenastrum capricornutum* and toxic strain *Microcystis aeruginosa* to exudates from two *Potamogeton species*. *Polish J. Environ. Stud.* 20 1359–1366.

[B55] Zhu S. S. Liu X. L. Liu P. F. Li J. Q. Wang H. M. Yuan S. K. (2007). Flumorph is a novel fungicide that disrupts microfilament organization in *Phytophthora melonis*. *Phytopathology* 97 643–649. 10.1094/PHYTO-97-5-0643 18943584

