# Corrigendum: Tobacco Rotated with Rapeseed for Soil-Borne *Phytophthora* Pathogen Biocontrol: Mediated by Rapeseed Root Exudates

**DOI:** 10.3389/fmicb.2018.00372

**Published:** 2018-02-26

**Authors:** Yuting Fang, Limeng Zhang, Yongge Jiao, Jingjing Liao, Lifen Luo, Sigui Ji, Jiangzhou Li, Kuai Dai, Shusheng Zhu, Min Yang

**Affiliations:** ^1^State Key Laboratory for Conservation and Utilization of Bio-Resources in Yunnan, Yunnan Agricultural University, Kunming, China; ^2^Key Laboratory for Agro-biodiversity and Pest Control of Ministry of Education, Yunnan Agricultural University, Kunming, China; ^3^Yunnan Tobacco Company, Yuxi Branch, Yuxi, China

**Keywords:** *Phytophthora parasitica*, root exudates, chemical interaction, soil-borne pathogen, rotation

In the published article, there was an error in affiliation (1). Instead of “State Key Laboratory for Conservation and Utilization of Bio-Resources in Yunnan, Yunnan University, Kunming, China”, it should be “State Key Laboratory for Conservation and Utilization of Bio-Resources in Yunnan, Yunnan Agricultural University, Kunming, China”. The authors apologize for this error and state that this does not change the scientific conclusions of the article in any way.

The original article has been updated.

## Conflict of interest statement

The authors declare that the research was conducted in the absence of any commercial or financial relationships that could be construed as a potential conflict of interest.

