# Supplementary material for: Tobacco Rotated with Rapeseed for Soil-Borne Phytophthora Pathogen Biocontrol: Mediated by Rapeseed Root Exudates
Source: Front Microbiol. 2016 Jun 13;7:894. doi: 10.3389/fmicb.2016.00894 (PMC4904020; doi:10.3389/fmicb.2016.00894)

Supplementary Material

Tobacco Rotated with Rapeseed for Soil-borne *Phytophthora* Pathogen Biocontrol: Mediated by Rapeseed Root Exudates

Yuting Fang [1,2#], Limeng Zhang[3#], Yongge Jiao[3#], Jingjing Liao[1,2], Lifen Luo[1,2], Sigui Ji[3], Jiangzhou Li[3], Kuai Dai[3], Shusheng Zhu[1,2]*, Min Yang[1,2]*

*Corresponding authors: Min Yang (yangminscnc@126.com); Shusheng Zhu (shushengzhu79@ 126.com)

Figure S1 Total ion chromatograms obtained using GC-MS for rapeseed root exudate.

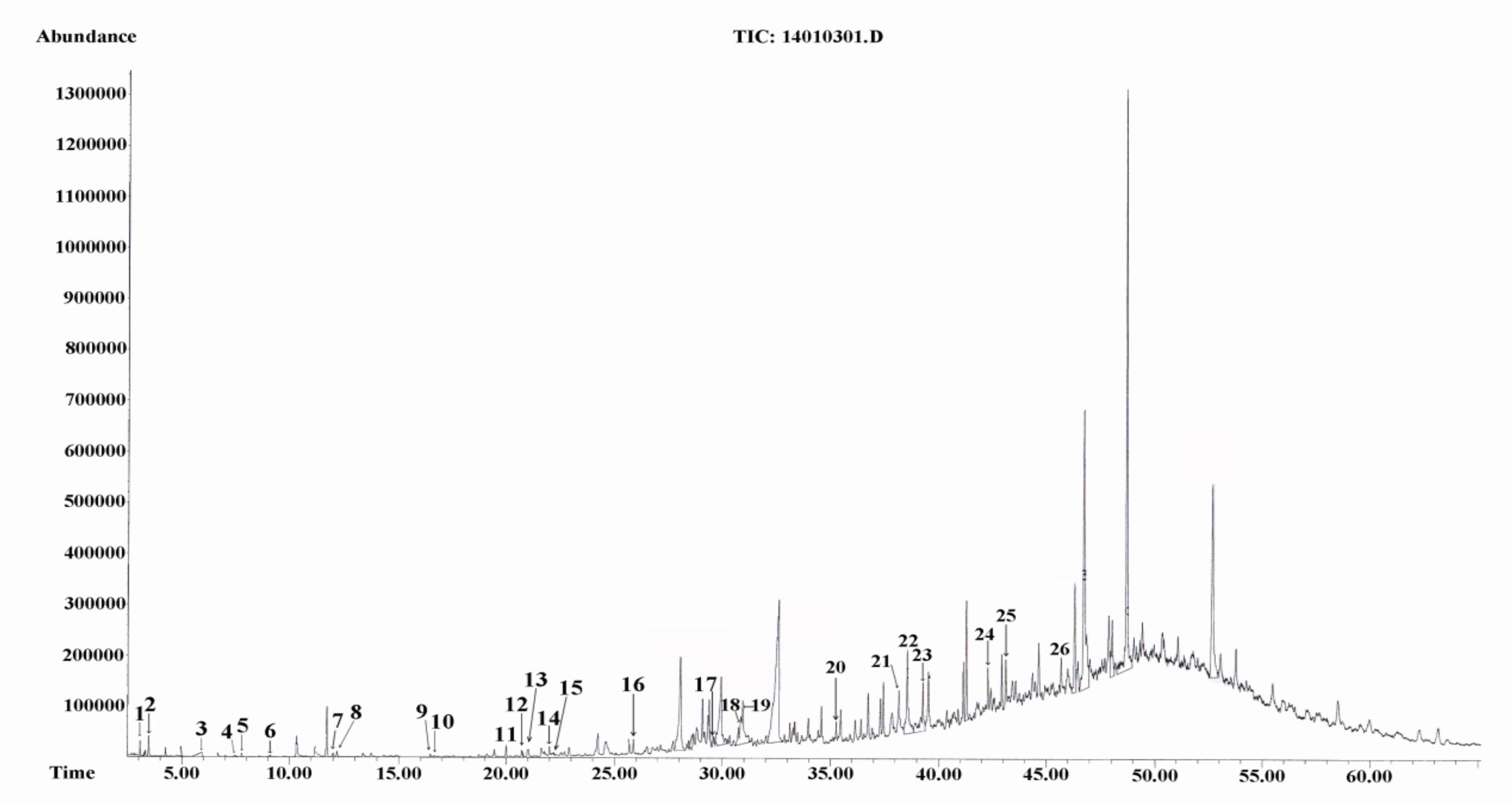

Figure S2. HPLC profiles for analytical standards (A) and root exudates of rapeseed (B). Identified compounds include: 1, 2-Butenoic acid; 2, benzothiazole; 3, 4-methoxyindole; 4, 1-(4- ethylphenyl)-ethanone; 5, 2-(methylthio)benzothiazole.

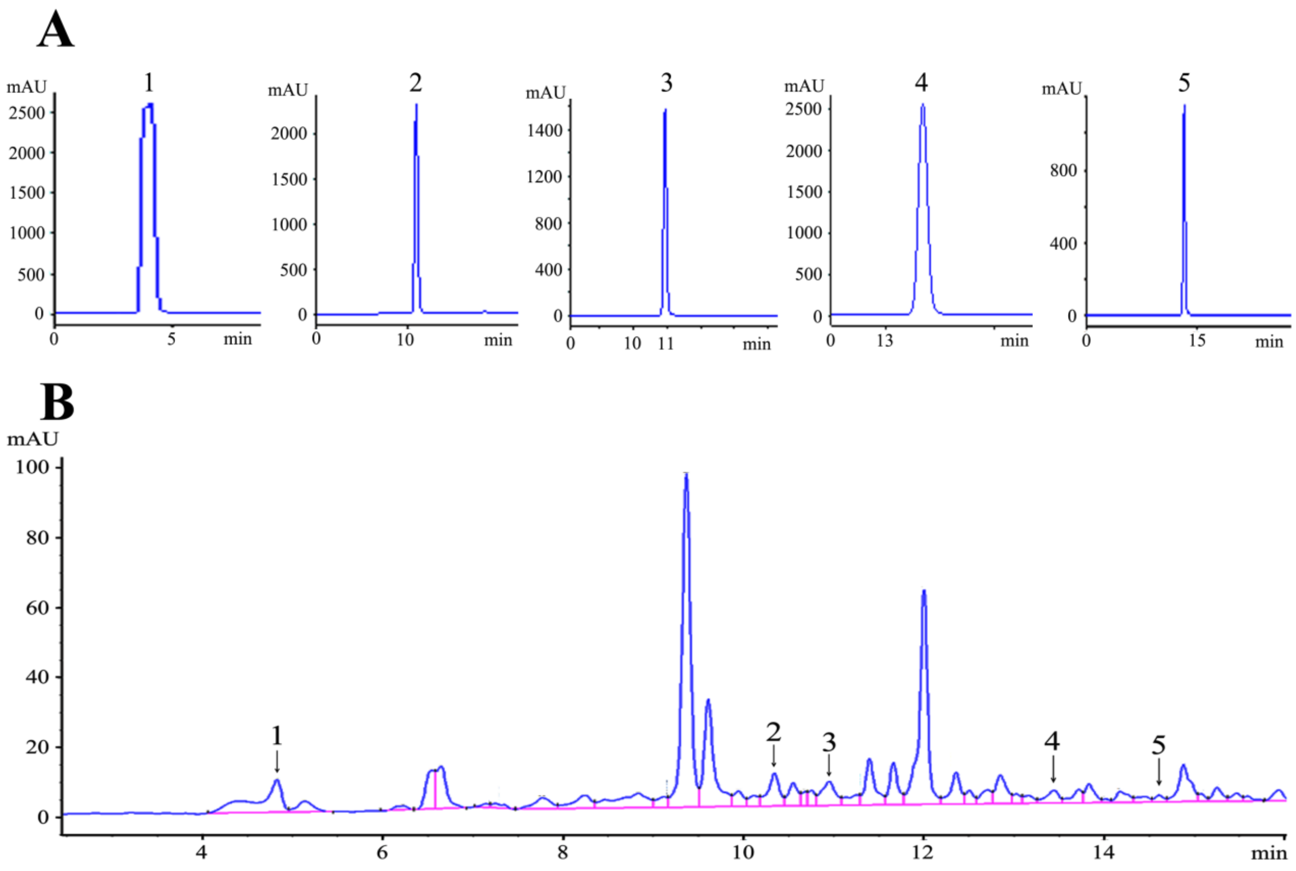

Supplement: Supplementary file 1 [file Image_1.PDF]
