# Supplementary material for: Tobacco Rotated with Rapeseed for Soil-Borne Phytophthora Pathogen Biocontrol: Mediated by Rapeseed Root Exudates
Source: Front Microbiol. 2016 Jun 13;7:894. doi: 10.3389/fmicb.2016.00894 (PMC4904020; doi:10.3389/fmicb.2016.00894)
Supplement: Supplementary file 2 [file Table_1.DOCX]

**Supplementary Material**

**Tobacco Rotated with Rapeseed for Soil-borne *Phytophthora* Pathogen Biocontrol: Mediated by Rapeseed Root Exudates**

Yuting Fang ^1,2#^, Limeng Zhang^3#^, Yongge Jiao^3#^, Jingjing Liao^1,2^, Lifen Luo^1,2^, Sigui Ji^3^, Jiangzhou Li^3^, Kuai Dai^3^, Shusheng Zhu^1,2^*, Min Yang^1,2^*

*Corresponding authors: Min Yang ([yangminscnc@126.com](mailto:yangminscnc@126.com)); Shusheng Zhu (shushengzhu79@ 126.com)

**Table S1** Effect of pure compounds identified by GC-MS on the mycelium growth of *P. parasitica* var. *nicotianae*

| Treatment | Rate of inhibition ± SD% | | | | EC_50_^a^  (mg/L) |
| --- | --- | --- | --- | --- | --- |
| 2-butenoic acid | 250 mg/L | 200 mg/L | 100 mg/L | 50 mg/L |  |
|  | 89.87±0.94a | 61.85±0.73b | 48.06±1.10c | 7.11±0.71d | 124.51 |
| Valeric acid | 350 mg/L | 200 mg/L | 150 mg/L | 20 mg/L |  |
|  | 89.12±0.41a | 61.19±1.79b | 37.99±1.67c | 20.12±2.14d | 118.37 |
| 4-Methoxyindole | 300 mg/L | 150 mg/L | 80 mg/L | 10 mg/L |  |
|  | 100.00±0.00a | 81.31±1.00b | 57.08±0.38c | 30.60±0.41d | 27.46 |
| Cyclohexyl isocyanate | 1000 mg/L | 600 mg/L | 400 mg/L | 200 mg/L |  |
|  | 77.82±1.47a | 57.70±1.67b | 49.08±1.96c | 37.99±0.83d | 366.37 |
| Benzothiazole | 300 mg/L | 200 mg/L | 100 mg/L | 10 mg/L |  |
|  | 73.19±3.35a | 41.95±3.36b | 15.72±2.30c | 4.73±1.47d | 228.81 |
| 2-(Methylthio)  benzothiazole | 300 mg/L | 200 mg/L | 100 mg/L | 50 mg/L |  |
|  | 97.99±0.55a | 95.77±0.80a | 47.69±3.05b | 12.47±1.27c | 93.86 |
| 1-(4-ethylphenyl)-ethanone | 700 mg/L | 400 mg/L | 200 mg/L | 100 mg/L |  |
|  | 100.00±0.00a | 75.25±1.79b | 27.57±1.53c | 13.48±0.71d | 205.53 |
| 2-Heptanone | 1000 mg/L | 800 mg/L | 600 mg/L | 400 mg/L |  |
|  | 6.89±2.98a | 10.86±1.50a | 6.68±0.78a | 3.76±1.76a | —^b^ |
| Decane | 1000 mg/L | 800 mg/L | 600 mg/L | 400 mg/L |  |
|  | 28.68±1.17a | 21.51±1.10b | 24.42±1.37ab | 21.71±0.71b | — |
| Undecane | 1000 mg/L | 800 mg/L | 600 mg/L | 400 mg/L |  |
|  | 12.02±1.45a | 13.37±0.79a | 6.01±0.81b | 1.55±0.39c | — |
| Dodecane | 1000 mg/L | 800 mg/L | 600 mg/L | 400 mg/L |  |
|  | -1.94±0.57c | 5.23±0.83a | 1.94±0.94b | -3.68±0.53c | — |
| Tetradecane | 1000 mg/L | 800 mg/L | 600 mg/L | 400 mg/L |  |
|  | 21.51±0.97a | -3.49±0.99b | -4.26±0.49b | -5.23±1.17b | — |
| Octadecane | 1000 mg/L | 800 mg/L | 600 mg/L | 400 mg/L |  |
|  | 3.88±1.08b | 13.57±0.57a | 10.66±0.36a | 1.55±0.79b | — |
| n-Hexadecanoic acid | 1000 mg/L | 800 mg/L | 600 mg/L | 400 mg/L |  |
|  | 15.89±0.57a | 14.92±1.08a | 14.73±1.10a | 18.60±1.26a | — |

^a^Concentration required to reduce growth by 50%; ^b^EC_50_ cannot be calculated due to their low antimicrobial activity.

**Table S2** The chemotaxis ratio of *P. parasitica* var. *nicotianae* zoospores to rapeseed roots

| min | Chemotaxis Ratio (CR)^a^ | |
| --- | --- | --- |
|  | Root tip | Root hairs |
| 1 | 1.73±0.41b | 2.19±0.57b |
| 5 | 4.19±1.27a | 4.95±0.93a |
| 10 | 4.05±1.48a | 5.44±1.60a |
| 15 | 3.74±1.20a | 4.93±4.54a |
| 20 | 6.58±4.89a | 4.68±4.32a |
| 25 | 2.11±0.78a | 3.78±0.81a |

^a^The chemotactic ratio (CR) = (scores of zoospores and cystospores on the test root)/(score of zoospores and cystospores on the control). Positive CR values indicate positive chemotaxis.
